# Extended Thermodynamics of Rarefied Polyatomic Gases: 15-Field Theory Incorporating Relaxation Processes of Molecular Rotation and Vibration

**DOI:** 10.3390/e20040301

**Published:** 2018-04-20

**Authors:** Takashi Arima, Tommaso Ruggeri, Masaru Sugiyama

**Affiliations:** 1 Department of Mechanical Engineering, Faculty of Engineering, Kanagawa University, Yokohama 221-8686, Japan; 2 Alma Mater Research Center on Applied Mathematics (AM^2^), Department of Mathematics, University of Bologna, Bologna 40123-I, Italy; 3 Graduate School of Engineering, Nagoya Institute of Technology, Nagoya 466-8555, Japan

**Keywords:** extended thermodynamics, rarefied polyatomic gas, relaxation process, molecular rotation and vibration, generalization of Navier–Stokes and Fourier theory

## Abstract

After summarizing the present status of Rational Extended Thermodynamics (RET) of gases, which is an endeavor to generalize the Navier–Stokes and Fourier (NSF) theory of viscous heat-conducting fluids, we develop the molecular RET theory of rarefied polyatomic gases with 15 independent fields. The theory is justified, at mesoscopic level, by a generalized Boltzmann equation in which the distribution function depends on two internal variables that take into account the energy exchange among the different molecular modes of a gas, that is, translational, rotational, and vibrational modes. By adopting the generalized Bhatnagar, Gross and Krook (BGK)-type collision term, we derive explicitly the closed system of field equations with the use of the Maximum Entropy Principle (MEP). The NSF theory is derived from the RET theory as a limiting case of small relaxation times via the Maxwellian iteration. The relaxation times introduced in the theory are shown to be related to the shear and bulk viscosities and heat conductivity.

## 1. Introduction

The Navier–Stokes and Fourier (NSF) theory, which was conceived nearly two centuries ago, has been recognized as the distinguished theory for describing viscous flow and heat conduction in a fluid. Its practical usefulness has also been repeatedly confirmed, in particular, in the field of engineering; mechanical engineering, aerospace engineering, chemical engineering, and so on.

The nonequilibrium-thermodynamical basis of the NSF theory was laid down by thermodynamics of irreversible processes (TIP) in the middle of the last century [1]. It is well known that TIP is valid for phenomena where the assumption of local thermal equilibrium is well satisfied. Therefore the NSF theory has the same limitation of its validity range. In other words, the NSF theory is no longer valid for describing phenomena beyond local equilibrium. Indeed, there exist many experimental evidences that the NSF theory becomes to be insufficient for highly nonequilibrium phenomena, for example, in shock waves and ultrasonic waves [2,3,4,5]. Generalization of the NSF theory is an urgent problem nowadays from both theoretical and practical viewpoints.

Several attempts have been done in order to overcome TIP. These are, for example, Extended Irreversible Thermodynamics [6,7], Rational Extended Thermodynamics [8,9], and GENERIC, which is an acronym for General Equation for Non-Equilibrium Reversible-Irreversible Coupling [10,11,12,13,14]. A first tentative of comparison among different approaches of nonequilibrium thermodynamics has been made in [15]. Rational Extended Thermodynamics (RET) of gases [8,9], in particular, has been established as a theory to explore highly nonequilibrium phenomena mentioned above, which utilizes the kinetic theory as its justification at mesoscopic level. Interplay between these theories and the numerical simulations using multiscale methods (see, for example [16,17]) is also quite interesting.

In the present paper, after presenting a brief review on the present status of RET of gases in the next section, we will establish a 15-field RET theory of rarefied polyatomic gases with two relaxation processes in which molecular rotation and vibration are involved. We will see that the NSF theory is deduced from the RET theory in the limit of small relaxation times.

## 2. Rational Extended Thermodynamics: Present Status

In RET, we adopt dissipative fluxes, such as viscous stress and heat flux, as independent variables in addition to the usual hydrodynamic variables, and we assume a system of balance equations with local-type constitutive equations. For rarefied gases, there are two possible closure methods for a system of balance equations:

- *Phenomenological RET*: The closure is obtained by using the universal principles of continuum thermomechanics—(I) *the Galilean invariance and the objectivity principle*; (II) *the entropy principle*; and (III) *the causality and thermodynamic stability (i.e., convexity of the entropy)*—to select admissible constitutive equations (see [8,18] for monatomic gases and [9,19] for polyatomic ones);
- *Molecular Extended Thermodynamics (molecular ET)*: The fields are the moments of a distribution function and the closure is done by using the *maximum entropy principle (MEP)* . This principle has its root in statistical mechanics. It is developed by Jaynes [20,21] in the context of the theory of information basing on the Shannon entropy. Nowadays the importance of MEP is recognized fully due to the numerous applications in many fields [22], for example, in the field of computer graphics. MEP states that the probability distribution that represents the current state of knowledge in the best way is the one with the largest entropy. Concerning the applicability of MEP in nonequilibrium thermodynamics, this was originally raised by the observation made by Kogan [23] that Grad’s distribution [24] function maximizes the entropy. The MEP was proposed in RET for the first time by Dreyer [25]. The MEP procedure was then generalized by Müller and Ruggeri to the case of any number of moments in the first edition of 1993 of their book [8] proving that the closed system is symmetric hyperbolic and coining the name of *molecular ET* for this kind of closure process.

It was proved that the closure by the MEP is equivalent to the imposition of the entropy principle on the truncated moment equations both for monatomic gases [26] and for polyatomic gases [27]. It was verified that the two closure methods for processes near equilibrium are also equivalent to the Grad kinetic closure based on the perturbation around the Maxwellian via Hermite polynomials [24,28] (see [8,25] for monatomic gases with 13 fields and [9,29] for polyatomic gases with 14 fields).

In order to generalize the NSF theory, it is suitable to study the following four cases individually: (i) rarefied monatomic gas; (ii) rarefied polyatomic gas; (iii) dense monatomic gas; and (iv) dense polyatomic gas; even though, in principle, the case (iv) includes all the other three cases as special ones.

### 2.1. RET of Rarefied Monatomic Gases

We adopt the system of balance equations with hierarchy structure, which is suggested by the moment theory based on the Boltzmann equation [8]. A well-known example is the Grad moment theory [24] although the phenomenological independent variables are not the moments of the distribution function. The hierarchy is characterized by: (i) the tensorial rank of the balance equations increases one by one starting from the mass balance equation; and (ii) the flux in one equation becomes the density in the next equation. The prototype of the phenomenological RET theory is the 13-field theory that adopts the mass density, velocity, internal energy, shear stress, and heat flux as independent fields [18]. The relativistic RET theory has also been established [30]. Furthermore, *molecular ET* of monatomic gases has been developed comprehensively [8,25].

### 2.2. RET of Rarefied Polyatomic Gases

The RET theory of rarefied polyatomic gases was developed in [9,19,31]. This theory adopts the balance equations with binary hierarchy structure, where the dynamic pressure (nonequilibrium pressure) is properly taken into account. Note that the dynamic pressure vanishes in rarefied monatomic gases. The number of independent fields is now 14; the mass density, velocity, internal energy, dynamic pressure, shear stress, and heat flux. The first hierarchy consists of the balance equations for mass density, momentum density and momentum flux, and the second hierarchy consists of the balance equations for energy density and energy flux.

The binary hierarchy was first introduced in a previous simple model [32] and in a kinetic approach [33], and, for the first time, in the phenomenological 14-field theory (ET${}_{14}$ theory) [19]. The hierarchy was justified and also derived from molecular ET [29] by using the generalized kinetic theory where the distribution function depends also on an extra variable that represents the molecular internal degrees of freedom such as rotation and vibration [34,35]. In a different context, the necessity to enlarge the kinetic theory of particles, that is, from rigid particles to particles with internal degrees of freedom, was discussed in [36].

The molecular ET theory of rarefied polyatomic gases with any number of fields was established in [27], and the convergence to the singular limit of a monatomic gas when the molecular degrees of freedom D→3 was proved in [37].

The validity of the ET${}_{14}$ theory of rarefied polyatomic gases has been successfully confirmed by comparing the theoretical predictions to the experimental data of linear waves [38], shock waves [39,40], and light scattering [41] in the region where the NSF theory is no longer valid.

The role of the dynamic pressure has been studied, in particular, by the RET theory with 6 fields (ET${}_{6}$): mass density, velocity, specific internal energy, and dynamic pressure [42,43]. This theory is the simplest extension of the Euler theory and is compatible with the Meixner theory with one internal variable [44,45]. The correspondence relation between ET${}_{6}$ and the Meixner theory was shown explicitly in [42]. Recently the ET${}_{6}$ theory with a nonlinear constitutive equation was proposed and developed [46,47,48,49]. It is also noteworthy that the molecular ET${}_{6}$ theory of rarefied polyatomic gases is consistent with a different approach of polyatomic gases via the discrete internal energy in which a gas is treated as a sort of mixture of monatomic gases [49]. It has been shown that the effect of the dynamic pressure becomes to be several orders of magnitude larger in some gases, e.g., hydrogen gas [9]. This fact is remarkable because the dynamic pressure is usually related to the bulk viscosity under the assumption of the Newtonian fluid, and sometimes it is assumed to be zero (Stokes’ assumption). The RET theory with two molecular relaxation processes was also developed by adopting 7 independent fields [50].

### 2.3. RET of Dense Gases

RET of dense gases is still under development [51,52,53,54]. In this case, we can rely solely on the phenomenological RET theory. Nonequilibrium properties of dense gases and liquids can be analyzed by this theory. Unfortunately, we cannot go into the details of this subject in the present paper because we will focus our attention on rarefied polyatomic gases in the next section.

## 3. RET Theory with Molecular Internal Variables

### 3.1. Molecular Internal Variables

Concerning the kinetic counterpart of the phenomenological ET${}_{14}$ theory of a rarefied polyatomic gas [19], a crucial step was made by Borgnakke and Larsen [34]. The distribution function is assumed to depend on an additional continuous variable *I* representing the energy of the internal modes of a molecule, such as molecular rotational and vibrational modes. As only one variable *I* is introduced here, the model treats the internal modes *as a unit*. Then the system of balance equations in RET is prescribed by two kinds of fields, that is, the fields related to the translational motion of a molecule and the fields related to the internal motion of a molecule. For this reason, this theory adopts the system of balance equations with binary hierarchy structure [27,29,42].

However, in the case when we need a more detailed model that describes the relaxation processes of rotational and vibrational modes *individually*, we must introduce two non-negative energies of the internal modes of a molecule, that is, the energy of rotational mode $I^{R}$ and the energy of vibrational mode $I^{V}$ [50]. Then we have the relationship:

$$\begin{array}{l} I=I^{R}+I^{V}. \end{array}$$

In this model, the velocity distribution function *f* depends also on the two parameters, i.e., $f\equiv f\left(x,c,t,I^{R},I^{V}\right)$, where $f(x,c,t,I^{R},I^{V})\;dx\;dc$ is the number density of molecules with the energies $I^{R}$ and $I^{V}$ at time *t* and in the volume element dxdc of the phase space (6D position-velocity space) centered at $\left(x,c\right)\in R^{3}\times R^{3}$. The Boltzmann equation is formally the same as the one of monatomic gases:

(1) $$\partial_{t}f+c_{i}\;\partial_{i}f=Q\left(f\right),$$

but, for the collision term Q(f), we take into account the influence of internal degrees of freedom through the collision cross-section. Here $\partial_{t}\equiv \partial /\partial t$ and $\partial_{i}\equiv \partial /\partial x_{i}$.

One remark is made here: In a harmonic approximation of the molecular vibration, we may divide $I^{V}$ into the energies of several harmonic modes, and may establish a model with several *I*’s. However, we do not enter into such details here because the extension in this direction is straightforward.

### 3.2. System of Balance Equations with Triple Hierarchy

Let us introduce three kinds of moments F, $H^{R}$, and $H^{V}$ as follows:

(2) $$\begin{array}{l} \begin{array}{l} F=\int_{R^{3}}\int_{0}^{\infty }\int_{0}^{\infty }mf\;\varphi \left(I^{R}\right)\psi \left(I^{V}\right)\;dI^{R}dI^{V}dc,\;\;\;\;\;\;\;\;F_{i_{1}\ldots i_{j}}=\int_{R^{3}}\int_{0}^{\infty }\int_{0}^{\infty }mc_{i_{1}}\cdots c_{i_{j}}f\;\varphi \left(I^{R}\right)\psi \left(I^{V}\right)\;dI^{R}dI^{V}dc,\\ H_{ll}^{R}=\int_{R^{3}}\int_{0}^{\infty }\int_{0}^{\infty }2I^{R}f\;\varphi \left(I^{R}\right)\psi \left(I^{V}\right)\;dI^{R}dI^{V}dc,\;H_{lli_{1}\ldots i_{k}}^{R}=\int_{R^{3}}\int_{0}^{\infty }\int_{0}^{\infty }2I^{R}c_{i_{1}}\cdots c_{i_{k}}f\;\varphi \left(I^{R}\right)\psi \left(I^{V}\right)\;dI^{R}dI^{V}dc,\\ H_{ll}^{V}=\int_{R^{3}}\int_{0}^{\infty }\int_{0}^{\infty }2I^{V}f\;\varphi \left(I^{R}\right)\psi \left(I^{V}\right)\;dI^{R}dI^{V}dc,\;H_{lli_{1}\ldots i_{l}}^{V}=\int_{R^{3}}\int_{0}^{\infty }\int_{0}^{\infty }2I^{V}c_{i_{1}}\cdots c_{i_{l}}f\;\varphi \left(I^{R}\right)\psi \left(I^{V}\right)\;dI^{R}dI^{V}dc, \end{array} \end{array}$$

where j,k,l=1,2,⋯. Here *m* is the mass of a molecule, and $\varphi \left(I^{R}\right)$ and $\psi \left(I^{V}\right)$ are the state densities corresponding to $I^{R}$ and $I^{V}$, i.e., $\varphi \left(I^{R}\right)dI^{R}$ ($\psi \left(I^{V}\right)dI^{V}$) represents the number of internal rotational (vibrational) state between $I^{R}$ and $I^{R}+dI^{R}$ ($I^{V}$ and $I^{V}+dI^{V}$). From the Boltzmann equation (1), we obtain three hierarchies (a triple hierarchy) of balance equations, in the form:

(3) $$\begin{array}{lll} \partial_{t}F+\partial_{i}F_{i}=0,\\ \partial_{t}F_{i_{1}}+\partial_{i}F_{ii_{1}}=0,\\ \partial_{t}F_{i_{1}i_{2}}+\partial_{i}F_{ii_{1}i_{2}}=P_{i_{1}i_{2}}^{K}, & \partial_{t}H_{ll}^{R}+\partial_{i}H_{lli}^{R}=P_{ll}^{R}, & \partial_{t}H_{ll}^{V}+\partial_{i}H_{lli}^{V}=P_{ll}^{V},\\ \partial_{t}F_{i_{1}i_{2}i_{3}}+\partial_{i}F_{ii_{1}i_{2}i_{3}}=P_{i_{1}i_{2}i_{3}}^{K}, & \partial_{t}H_{lli_{1}}^{R}+\partial_{i}H_{llii_{1}}^{R}=P_{lli_{1}}^{R}, & \partial_{t}H_{lli_{1}}^{V}+\partial_{i}H_{llii_{1}}^{V}=P_{lli_{1}}^{V},\\ \;\;\vdots & \;\;\vdots & \;\;\vdots \end{array}$$

where the production terms are related to the collision term as follows:

(4) $$\begin{array}{l} \begin{array}{l} P_{i_{1}\ldots i_{j}}^{K}=\int_{R^{3}}\int_{0}^{\infty }\int_{0}^{\infty }mc_{i_{1}}\cdots c_{i_{j}}Q\left(f\right)\;\varphi \left(I^{R}\right)\psi \left(I^{V}\right)\;dI^{R}dI^{V}dc,\\ P_{lli_{1}\ldots i_{k}}^{R}=\int_{R^{3}}\int_{0}^{\infty }\int_{0}^{\infty }2I^{R}c_{i_{1}}\cdots c_{i_{k}}Q\left(f\right)\;\varphi \left(I^{R}\right)\psi \left(I^{V}\right)\;dI^{R}dI^{V}dc,\\ P_{lli_{1}\ldots i_{l}}^{V}=\int_{R^{3}}\int_{0}^{\infty }\int_{0}^{\infty }2I^{V}c_{i_{1}}\cdots c_{i_{l}}Q\left(f\right)\;\varphi \left(I^{R}\right)\psi \left(I^{V}\right)\;dI^{R}dI^{V}dc. \end{array} \end{array}$$

We notice that the first and second equations of the *F*-hierarchy represent the conservation laws of mass and momentum, while the sum of the balance equations of $F_{ll}$, $H_{ll}^{R}$ and $H_{ll}^{V}$ represents the conservation law of energy:

$$\begin{array}{l} \partial_{t}G_{ll}+\partial_{i}G_{ill}=0, \end{array}$$

with

$$\begin{array}{l} G_{ll}=F_{ll}+H_{ll}^{R}+H_{ll}^{V},\;G_{ill}=F_{ill}+H_{ill}^{R}+H_{ill}^{V}, \end{array}$$

and the production term $Q_{ll}$, which vanishes:

$$\begin{array}{l} Q_{ll}=P_{ll}^{K}+P_{ll}^{R}+P_{ll}^{V}=0. \end{array}$$

It is remarkable that, in each of the three hierarchies, the flux in one equation appears as the density in the next equation.

### 3.3. Entropy Law

The entropy density *h*, the entropy flux $h_{i}$, and the entropy production Σ are defined by

(5) $$\begin{array}{l} \begin{array}{l} h=-k_{B}\int_{R^{3}}\int_{0}^{\infty }\int_{0}^{\infty }f\log f\;\varphi \left(I^{R}\right)\psi \left(I^{V}\right)\;dI^{R}dI^{V}dc,\\ h_{i}=-k_{B}\int_{R^{3}}\int_{0}^{\infty }\int_{0}^{\infty }c_{i}f\log f\;\varphi \left(I^{R}\right)\psi \left(I^{V}\right)\;dI^{R}dI^{V}dc,\\ \Sigma =-k_{B}\int_{R^{3}}\int_{0}^{\infty }\int_{0}^{\infty }Q\left(f\right)\log f\;\varphi \left(I^{R}\right)\psi \left(I^{V}\right)\;dI^{R}dI^{V}dc, \end{array} \end{array}$$

where $k_{B}$ is the Boltzmann constant. Then we have the entropy law:

$$\begin{array}{l} \partial_{t}h+\partial_{i}h_{i}=\Sigma . \end{array}$$

The *H*-theorem requires that the collision term *Q* must be such that Σ given by the third equation in (5) is not negative: Σ≥0. We will see that the Bhatnagar, Gross and Krook (BGK) collision term, which will be proposed in Section 3.7.1, satisfies the *H*-theorem.

### 3.4. Equilibrium Distribution Function

We derive the equilibrium distribution function $f_{E}$ by means of MEP. We remark that the collision invariants of the present model are *m*, $mc_{i}$, and $mc^{2}+2I^{R}+2I^{V}$. These quantities correspond to the hydrodynamics variables, i.e., the mass density F(=ρ), the momentum density $F_{i}\left(=\rho v_{i}\right)$, and twice the energy density $G_{ll}\left(=2\rho \varepsilon +\rho v^{2}\right)$:

(6) $$\begin{array}{l} G_{ll}=\int_{R^{3}}\int_{0}^{\infty }\int_{0}^{\infty }\left(mc^{2}+2I^{R}+2I^{V}\right)f\;\varphi \left(I^{R}\right)\psi \left(I^{V}\right)\;dI^{R}dI^{V}dc. \end{array}$$

It is easy to see, from (6), that the specific internal energy ε is composed of the kinetic, rotational, and vibrational parts, $\varepsilon^{K}$, $\varepsilon^{R}$, and $\varepsilon^{V}$, i.e.,

$$\varepsilon =\varepsilon^{K}+\varepsilon^{R}+\varepsilon^{V}.$$

The equilibrium distribution function $f_{E}$, which maximizes the entropy density, the first equation in (5), under the constraints that the first 5 moments are prescribed, is given by

(7) $$\begin{array}{l} f_{E}=f_{E}^{\left(K\right)}f_{E}^{\left(R\right)}f_{E}^{\left(V\right)} \end{array}$$

with

$$\begin{array}{l} f_{E}^{\left(K\right)}=\frac{\rho }{m}{\left(\frac{m}{2\pi k_{B}T}\right)}^{3/2}\exp\left(-\frac{mC^{2}}{2k_{B}T}\right),\;f_{E}^{\left(R\right)}=\frac{1}{A^{R}\left(T\right)}\exp\left(-\frac{I^{R}}{k_{B}T}\right),\;f_{E}^{\left(V\right)}=\frac{1}{A^{V}\left(T\right)}\exp\left(-\frac{I^{V}}{k_{B}T}\right), \end{array}$$

where $A^{R}\left(T\right)$ and $A^{V}\left(T\right)$ are normalization factors (partition functions):

(8) $$\begin{array}{l} A^{R}\left(T\right)=\int_{0}^{\infty }\varphi \left(I^{R}\right)e^{-\beta_{E}I^{R}}dI^{R},\;A^{V}\left(T\right)=\int_{0}^{\infty }\psi \left(I^{V}\right)e^{-\beta_{E}I^{V}}dI^{V}. \end{array}$$

Here $\beta_{E}=1/\left(k_{B}T\right)$, *T* is the equilibrium temperature, and $C_{i}=c_{i}-v_{i}$ $\left(C^{2}=C_{j}C_{j}\right)$ is the peculiar velocity. For the proof see [46,49,50].

Using the equilibrium distribution function $f_{E}$, we obtain the caloric and thermal equations of state. The caloric equation of state is given by

(9) $$\begin{array}{l} \varepsilon =\varepsilon_{E}\left(T\right)=\varepsilon_{E}^{K}\left(T\right)+\varepsilon_{E}^{R}\left(T\right)+\varepsilon_{E}^{V}\left(T\right), \end{array}$$

and, in a similar way in [49], we have

(10) $$\begin{array}{l} \varepsilon_{E}^{K}\left(T\right)\equiv \frac{3}{2}\frac{k_{B}}{m}T,\;\varepsilon_{E}^{R}\left(T\right)\equiv \frac{k_{B}}{m}T^{2}\frac{d\log A^{R}\left(T\right)}{dT},\;\varepsilon_{E}^{V}\left(T\right)\equiv \frac{k_{B}}{m}T^{2}\frac{d\log A^{V}\left(T\right)}{dT}. \end{array}$$

If the partition functions $A^{R}$ and $A^{V}$ are given, for example, by a statistical-mechanical analysis, we obtain the equilibrium energies of rotational and vibrational modes from (10). Vice versa, if we know the caloric equations of state $\varepsilon_{E}^{R}\left(T\right)$ and $\varepsilon_{E}^{V}\left(T\right)$, we can obtain, by integration of the second and third equations in (10), the partition functions. We remark that the knowledge of the partition functions permits us to obtain, from (8), the measures $\varphi \left(I^{R}\right)$ and $\psi \left(I^{V}\right)$ via the inverse Laplace transformation. The thermal equation of state is given by

(11) $$\begin{array}{l} p=p^{K}\left(\rho ,T\right)\equiv \frac{k_{B}}{m}\rho T=\frac{2}{3}\rho \varepsilon_{E}^{K}\left(T\right), \end{array}$$

where *p* is the pressure.

The specific entropy density in equilibrium $s=h_{E}/\rho$ is given by

$$\begin{array}{l} s=s_{E}\left(\rho ,T\right)=s_{E}^{K}\left(\rho ,T\right)+s_{E}^{R}\left(T\right)+s_{E}^{V}\left(T\right), \end{array}$$

where

$$\begin{array}{ll} s_{E}^{K}\left(\rho ,T\right) & \equiv -\frac{k_{B}}{\rho }\int_{R^{3}}\int_{0}^{\infty }\int_{0}^{\infty }f_{E}\log f_{E}^{\left(K\right)}\;\varphi \left(I^{R}\right)\psi \left(I^{V}\right)\;dI^{R}dI^{V}dc,\\ & =\frac{k_{B}}{m}\log\left(\frac{T^{3/2}}{\rho }\right)+\frac{\varepsilon_{E}^{K}\left(T\right)}{T}-\frac{k_{B}}{m}\log\left[\frac{1}{m}{\left(\frac{m}{2\pi k_{B}}\right)}^{3/2}\right],\\ s_{E}^{R}\left(T\right) & \equiv -\frac{k_{B}}{\rho }\int_{R^{3}}\int_{0}^{\infty }\int_{0}^{\infty }f_{E}\log f_{E}^{\left(R\right)}\;\varphi \left(I^{R}\right)\psi \left(I^{V}\right)\;dI^{R}dI^{V}dc,\\ & =\frac{k_{B}}{m}\log A^{R}\left(T\right)+\frac{\varepsilon_{E}^{R}\left(T\right)}{T},\\ s_{E}^{V}\left(T\right) & \equiv -\frac{k_{B}}{\rho }\int_{R^{3}}\int_{0}^{\infty }\int_{0}^{\infty }f_{E}\log f_{E}^{\left(V\right)}\;\varphi \left(I^{R}\right)\psi \left(I^{V}\right)\;dI^{R}dI^{V}dc,\\ & =\frac{k_{B}}{m}\log A^{V}\left(T\right)+\frac{\varepsilon_{E}^{V}\left(T\right)}{T}. \end{array}$$

### 3.5. Molecular ET Theory with 7 Independent Fields (ET${}_{7}$)

The simplest RET theory with the triple hierarchy is the theory with 7 fields (ET${}_{7}$); mass density, velocity, internal energy of translational mode, internal energy of rotational mode, and internal energy of vibrational mode [50]. The system of balance equations is the system of (3) but truncated at the second order tensor:

$$\begin{array}{l} \begin{array}{l} \frac{\partial F}{\partial t}+\frac{\partial F_{i}}{\partial x_{i}}=0,\\ \frac{\partial F_{j}}{\partial t}+\frac{\partial F_{ij}}{\partial x_{i}}=0,\\ \frac{\partial F_{ll}}{\partial t}+\frac{\partial F_{lli}}{\partial x_{i}}=P_{ll}^{K},\;\frac{\partial H_{ll}^{R}}{\partial t}+\frac{\partial H_{lli}^{R}}{\partial x_{i}}=P_{ll}^{R},\;\frac{\partial H_{ll}^{V}}{\partial t}+\frac{\partial H_{lli}^{V}}{\partial x_{i}}=P_{ll}^{V}. \end{array} \end{array}$$

On the basis of this theory, it was revealed that the internal energies of the three modes can be characterized by the three nonequilibrium temperatures (see (14)), and the nonequilibrium entropy is expressed in terms of these nonequilibrium temperatures. It was also shown that the dispersion relation derived by ET${}_{7}$ is in excellent agreement with the experimental data of CO${}_{2}$, Cl${}_{2}$ and Br${}_{2}$ gases [50]. Moreover, this theory includes, as a limiting case, three different ET${}_{6}$ [50,53] theories, which correspond to different molecular relaxation processes. The previous ET${}_{6}$ theory discussed in Section 2.2 is, in fact, one of the three theories.

### 3.6. Molecular ET Theory with 15 Independent Fields (ET${}_{15}$)

The ET${}_{7}$ theory describes the relaxation processes of molecular rotational and vibrational modes satisfactorily, but it ignores the effect of shear stress and heat flux. In this section, we establish and develop more realistic theory including all these dissipative fluxes.

As we will see in (18), the shear stress is the intrinsic (velocity-independent) part of the traceless momentum flux $F_{\langle ij\rangle }$ and the (total) heat flux is the intrinsic part of the total energy flux $G_{lli}\left(=F_{lli}+H_{lli}^{R}+H_{lli}^{V}\right)$. We, therefore, study the truncated system of (3) with the densities *F*, $F_{i}$, $F_{ll}$, $H_{ll}^{R}$, $H_{ll}^{V}$, $F_{\langle ij\rangle }$ and $G_{lli}$, which we call ET${}_{15}$ theory:

(12) $$\begin{array}{l} \begin{array}{l} \frac{\partial F}{\partial t}+\frac{\partial F_{i}}{\partial x_{i}}=0,\\ \frac{\partial F_{j}}{\partial t}+\frac{\partial F_{ij}}{\partial x_{i}}=0,\\ \frac{\partial F_{ij}}{\partial t}+\frac{\partial F_{ijk}}{\partial x_{k}}=P_{ij}^{K},\;\;\;\frac{\partial H_{ll}^{R}}{\partial t}+\frac{\partial H_{lli}^{R}}{\partial x_{i}}=P_{ll}^{R},\;\frac{\partial H_{ll}^{V}}{\partial t}+\frac{\partial H_{lli}^{V}}{\partial x_{i}}=P_{ll}^{V},\\ \;\;\;\;\;\;\;\;\;\frac{\partial G_{lli}}{\partial t}+\frac{\partial G_{llik}}{\partial x_{k}}=Q_{lli}, \end{array} \end{array}$$

where ($F_{ijk}$, $H_{lli}^{R}$, $H_{lli}^{V}$, $G_{llik}$) and ($P_{ij}^{K}$, $P_{ll}^{R}$, $P_{ll}^{V}$, $Q_{lli}$) are, respectively, the fluxes and productions of the densities ($F_{ij}$, $H_{ll}^{R}$, $H_{ll}^{V}$, $G_{lli}$). It should be noted that, the balance equation of the energy flux $G_{lli}$ is obtained by summing the balance equations of $F_{lli}$, $H_{lli}^{R}$, and $H_{lli}^{V}$ that appear in (3):

$$\begin{array}{l} G_{lli}=\int_{R^{3}}\int_{0}^{\infty }\int_{0}^{\infty }\left(mc^{2}+2I^{R}+2I^{V}\right)c_{i}\;f\;\varphi \left(I^{R}\right)\psi \left(I^{V}\right)\;dI^{R}dI^{V}dc. \end{array}$$

Then we have

$$\begin{array}{l} G_{llik}=F_{llik}+H_{llik}^{R}+H_{llik}^{V},\;Q_{lli}=P_{lli}^{K}+P_{lli}^{R}+P_{lli}^{V}. \end{array}$$

It is worth emphasizing again that the ET${}_{14}$ theory adopts $F,F_{i},F_{ij},G_{ll}\left(=F_{ll}+H_{ll}^{R}+H_{ll}^{V}\right)$ and $G_{lli}$ as independent fields and the internal modes of a molecule are treated as a unit. On the other hand, ET${}_{15}$ describes the rotational and vibrational modes individually.

#### 3.6.1. Galilean Invariance and Intrinsic Variables

The densities of the system (12) are related to the following conventional field variables:

(13) $$\begin{array}{ll} \mathrm{mass}\;\mathrm{density}: & \rho \equiv \int_{R^{3}}\int_{0}^{\infty }\int_{0}^{\infty }mf\;\varphi \left(I^{R}\right)\psi \left(I^{V}\right)\;dI^{R}dI^{V}dc,\\ \mathrm{velocity}: & v_{i}\equiv \frac{1}{\rho }\int_{R^{3}}\int_{0}^{\infty }\int_{0}^{\infty }mc_{i}f\;\varphi \left(I^{R}\right)\psi \left(I^{V}\right)\;dI^{R}dI^{V}dc,\\ \mathrm{specific}\;\mathrm{translational}\;\mathrm{energy}\;\mathrm{density}: & \varepsilon^{K}\equiv \frac{1}{2\rho }\int_{R^{3}}\int_{0}^{\infty }\int_{0}^{\infty }mC^{2}f\;\varphi \left(I^{R}\right)\psi \left(I^{V}\right)\;dI^{R}dI^{V}dc,\\ \mathrm{specific}\;\mathrm{rotational}\;\mathrm{energy}\;\mathrm{density}: & \varepsilon^{R}\equiv \frac{1}{\rho }\int_{R^{3}}\int_{0}^{\infty }\int_{0}^{\infty }mI^{R}f\;\varphi \left(I^{R}\right)\psi \left(I^{V}\right)\;dI^{R}dI^{V}dc,\\ \mathrm{specific}\;\mathrm{vibrational}\;\mathrm{energy}\;\mathrm{density}: & \varepsilon^{V}\equiv \frac{1}{\rho }\int_{R^{3}}\int_{0}^{\infty }\int_{0}^{\infty }mI^{V}f\;\varphi \left(I^{R}\right)\psi \left(I^{V}\right)\;dI^{R}dI^{V}dc,\\ \mathrm{shear}\;\mathrm{stress} & \sigma_{\langle ij\rangle }\equiv -\int_{R^{3}}\int_{0}^{\infty }\int_{0}^{\infty }mC_{\langle i}C_{j\rangle }f\;\varphi \left(I^{R}\right)\psi \left(I^{V}\right)\;dI^{R}dI^{V}dc,\\ \mathrm{heat}\;\mathrm{flux} & q_{i}\equiv \frac{1}{2}\int_{R^{3}}\int_{0}^{\infty }\int_{0}^{\infty }\left(mC^{2}+2I^{R}+2I^{V}\right)C_{i}f\;\varphi \left(I^{R}\right)\psi \left(I^{V}\right)\;dI^{R}dI^{V}dc. \end{array}$$

We define three new variables $\theta^{K}$, $\theta^{R}$, and $\theta^{V}$ associated with the specific energies $\varepsilon^{K}$, $\varepsilon^{R}$, and $\varepsilon^{V}$ in the third to fifth equations in (13) through the caloric equations of state given in (10) [50]:

(14) $$\begin{array}{l} \varepsilon^{K}=\varepsilon_{E}^{K}\left(\theta^{K}\right),\;\varepsilon^{R}=\varepsilon_{E}^{R}\left(\theta^{R}\right),\;\varepsilon^{V}=\varepsilon_{E}^{V}\left(\theta^{V}\right). \end{array}$$

Because of the monotonicity of $\varepsilon_{E}^{K,R,V}$, these are one-to-one relations between the new variables $\theta^{K,R,V}$ and the specific energies $\varepsilon^{K,R,V}$. The physical meaning of these parameters will be discussed in Section 3.6.3. From (7), the temperature *T* is determined in an implicit way by the relation:

(15) $$\begin{array}{l} \varepsilon_{E}\left(T\right)=\varepsilon_{E}^{K}\left(\theta^{K}\right)+\varepsilon_{E}^{R}\left(\theta^{R}\right)+\varepsilon_{E}^{V}\left(\theta^{V}\right). \end{array}$$

The trace part of the momentum flux is related to the pressure *p* and the dynamic pressure Π in continuum mechanics as follows:

$$\begin{array}{l} F_{ll}=3\left(p+\Pi \right)+\rho v^{2}. \end{array}$$

Therefore, from (2), (11), the third equation in (13), and (14), the dynamic pressure is expressed as [46,51]:

(16) $$\begin{array}{l} \Pi =p^{K}\left(\rho ,\theta^{K}\right)-p^{K}\left(\rho ,T\right)=\frac{2}{3}\rho \left(\varepsilon_{E}^{K}\left(\theta^{K}\right)-\varepsilon_{E}^{K}\left(T\right)\right). \end{array}$$

For later convenience, we introduce the following variables, i.e., energy deviations of the three modes from the state with the common temperature *T* [50,51,52,54]:

(17) $$\begin{array}{l} \begin{array}{l} \Delta^{K}=\varepsilon_{E}^{K}\left(\theta^{K}\right)-\varepsilon_{E}^{K}\left(T\right),\\ \Delta^{R}=\varepsilon_{E}^{R}\left(\theta^{R}\right)-\varepsilon_{E}^{R}\left(T\right),\\ \Delta^{V}=\varepsilon_{E}^{V}\left(\theta^{V}\right)-\varepsilon_{E}^{V}\left(T\right). \end{array} \end{array}$$

Then, from (16), the dynamic pressure Π is expressed as

$$\begin{array}{l} \Pi =\frac{2}{3}\rho \Delta^{K}. \end{array}$$

Since the intrinsic variables are the moments in terms of the peculiar velocity $C_{i}$ instead of $c_{i}$, we have in agreement with the Galilean invariance [55]:

(18) $$\begin{array}{l} \begin{array}{l} F=\rho ,\;F_{i}=\rho v_{i},\;F_{ll}=2\rho \varepsilon^{K}+\rho v^{2},\;F_{\langle ij\rangle }=-\sigma_{\langle ij\rangle }+\rho v_{\langle i}v_{j\rangle },\\ H_{ll}^{R}=2\rho \varepsilon^{R},\;H_{ll}^{V}=2\rho \varepsilon^{V},\\ G_{lli}=2q_{i}+\left\{2\left(\rho \varepsilon +p(\rho ,\theta^{K})\right)\right\}v_{i}-2\sigma_{\langle li\rangle }v_{l}+\rho v^{2}v_{i}. \end{array} \end{array}$$

Similarly, the velocity dependence of the fluxes and productions is obtained as follows:

$$\begin{array}{l} \begin{array}{l} F_{ijk}={\hat{F}}_{ijk}+3{\hat{F}}_{(ij}v_{k)}+\rho v_{i}v_{j}v_{k},\\ H_{lli}^{R}={\hat{H}}_{ll}^{R}v_{i}+{\hat{H}}_{lli}^{R},\;H_{lli}^{V}={\hat{H}}_{ll}^{V}v_{i}+{\hat{H}}_{lli}^{V},\\ G_{llik}={\hat{G}}_{llik}+2{\hat{G}}_{ll(i}v_{k)}+2v_{l}{\hat{F}}_{lik}+6v_{(i}v_{l}{\hat{F}}_{lk)}+v_{i}v_{k}\left({\hat{H}}_{ll}^{R}+{\hat{H}}_{ll}^{V}\right)+\rho v^{2}v_{i}v_{k},\\ P_{ij}^{K}={\hat{P}}_{ij}^{K},\;P_{ll}^{R}={\hat{P}}_{ll}^{R},\;P_{ll}^{V}={\hat{P}}_{ll}^{V},\\ Q_{lli}=2v_{l}{\hat{P}}_{il}^{K}+{\hat{Q}}_{lli}, \end{array} \end{array}$$

where a hat on a quantity indicates the intrinsic part of the quantity.

#### 3.6.2. Nonequilibrium Distribution Function Derived from MEP

To close the system (12), we need the nonequilibrium distribution function *f*, which is derived from the MEP. That is, the most suitable distribution function *f* of the truncated system (12) is obtained by maximizing the functional defined by

$$\begin{array}{l} L\left(f\right)\\ =-k_{B}\int_{R^{3}}\int_{0}^{\infty }\int_{0}^{\infty }f\log f\;\varphi \left(I^{R}\right)\psi \left(I^{V}\right)\;dI^{R}dI^{V}dc\\ \;\;\;+\lambda \left(F-\int_{R^{3}}\int_{0}^{\infty }\int_{0}^{\infty }mf\varphi \left(I^{R}\right)\psi \left(I^{V}\right)\;dI^{R}dI^{V}dc\right)+\lambda_{i}\left(F_{i}-\int_{R^{3}}\int_{0}^{\infty }\int_{0}^{\infty }mc_{i}f\varphi \left(I^{R}\right)\psi \left(I^{V}\right)\;dI^{R}dI^{V}dc\right)\\ \;\;\;+\lambda_{ij}\left(F_{ij}-\int_{R^{3}}\int_{0}^{\infty }\int_{0}^{\infty }mc_{i}c_{j}f\varphi \left(I^{R}\right)\psi \left(I^{V}\right)\;dI^{R}dI^{V}dc\right)\\ \;\;\;+\mu_{}^{R}\left(H_{ll}^{R}-\int_{R^{3}}\int_{0}^{\infty }\int_{0}^{\infty }2I^{R}f\varphi \left(I^{R}\right)\psi \left(I^{V}\right)\;dI^{R}dI^{V}dc\right)+\mu_{}^{V}\left(H_{ll}^{V}-\int_{R^{3}}\int_{0}^{\infty }\int_{0}^{\infty }2I^{V}f\varphi \left(I^{R}\right)\psi \left(I^{V}\right)\;dI^{R}dI^{V}dc\right)\\ \;\;\;+\mu_{i}\left(G_{lli}-\int_{R^{3}}\int_{0}^{\infty }\int_{0}^{\infty }mc^{2}c_{i}f\varphi \left(I^{R}\right)\psi \left(I^{V}\right)\;dI^{R}dI^{V}dc\right), \end{array}$$

where λ, $\lambda_{i}$, $\lambda_{ij}$, $\mu^{R}$, $\mu^{V}$, and $\mu_{i}$ are the Lagrange multipliers. Their velocity dependences are determined as follows [55]:

(19) $$\begin{array}{l} \begin{array}{l} \lambda =\hat{\lambda }-v_{i}{\hat{\lambda }}_{i}+v_{i}v_{j}{\hat{\lambda }}_{ij}-v^{2}v_{i}{\hat{\mu }}_{i},\\ \lambda_{i}={\hat{\lambda }}_{i}-2v_{j}{\hat{\lambda }}_{ji}+2v_{i}v_{j}{\hat{\mu }}_{j}+v^{2}{\hat{\mu }}_{i},\\ \lambda_{ij}={\hat{\lambda }}_{ij}-v_{l}{\hat{\mu }}_{l}\delta_{ij}-v_{i}{\hat{\mu }}_{j}-v_{j}{\hat{\mu }}_{i},\\ \mu^{R}={\hat{\mu }}^{R}-v_{i}{\hat{\mu }}_{i},\\ \mu^{V}={\hat{\mu }}^{V}-v_{i}{\hat{\mu }}_{i},\\ \mu_{i}={\hat{\mu }}_{i}. \end{array} \end{array}$$

The distribution function *f*, which satisfies δL/δf=0, is

(20) $$\begin{array}{l} \begin{array}{l} f_{15}=\exp\left(-1-\frac{m}{k_{B}}\chi \right),\\ \chi =\hat{\lambda }+C_{i}{\hat{\lambda }}_{i}+C_{i}C_{j}{\hat{\lambda }}_{ij}+\frac{2I^{R}}{m}{\hat{\mu }}^{R}+\frac{2I^{V}}{m}{\hat{\mu }}^{V}+\left(C^{2}+\frac{2I^{R}}{m}+\frac{2I^{V}}{m}\right)C_{i}{\hat{\mu }}_{i}. \end{array} \end{array}$$

Taking into account that, in equilibrium, $f_{15}$ coincides with the equilibrium distribution function (7), we can easily see that the equilibrium components of the Lagrange multipliers are given by

(21) $$\begin{array}{l} \lambda_{E}=-\frac{g}{T}+\frac{v^{2}}{2T},\;\lambda_{i_{E}}=-\frac{v_{i}}{T},\;\frac{\lambda_{ll_{E}}}{3}=\mu_{E}^{R}=\mu_{E}^{V}=\frac{1}{2T},\;\lambda_{{\langle ij\rangle }_{E}}=0,\;\mu_{i_{E}}=0, \end{array}$$

where *g* is the chemical potential. For the reason of non-convergence of the integrals in the nonlinear case (see [9,26] for a discussion on this subject), we consider, as usual, the processes near equilibrium. Then we expand (20) around an equilibrium state in the following form:

(22) $$\begin{array}{l} \begin{array}{l} f_{15}=f_{E}\left(1-\frac{m}{k_{B}}\tilde{\chi }\right),\\ \tilde{\chi }=\tilde{\lambda }+C_{i}{\tilde{\lambda }}_{i}+C_{i}C_{j}{\tilde{\lambda }}_{ij}+\frac{2I^{R}}{m}{\tilde{\mu }}^{R}+\frac{2I^{V}}{m}{\tilde{\mu }}^{V}+\left(C^{2}+\frac{2I^{R}}{m}+\frac{2I^{V}}{m}\right)C_{i}{\tilde{\mu }}_{i}, \end{array} \end{array}$$

where a tilde on a quantity indicates its nonequilibrium part. In the following, for simplicity, we use the notation *f* instead of $f_{15}$.

Inserting (22) into (13), we obtain the intrinsic nonequilibrium Lagrange multipliers as functions of $\left(\rho ,v_{i},T,\Delta^{R},\Delta^{V},\sigma_{\langle ij\rangle },q_{i}\right)$ up to the first order with respect to the nonequilibrium fields, $\Delta^{R}$, $\Delta^{V}$, $\sigma_{\langle ij\rangle }$ and $q_{i}$, as follows:

(23) $$\begin{array}{l} \begin{array}{l} \tilde{\lambda }=\frac{\varepsilon_{E}^{K}\left(T\right)}{T^{2}c_{v}^{K}\left(T\right)}\Delta^{K}+\frac{\varepsilon_{E}^{R}\left(T\right)}{T^{2}c_{v}^{R}\left(T\right)}\Delta^{R}+\frac{\varepsilon_{E}^{V}\left(T\right)}{T^{2}c_{v}^{V}\left(T\right)}\Delta^{V},\\ {\tilde{\lambda }}_{i}=\frac{\frac{k_{B}}{m}T+\varepsilon_{E}\left(T\right)}{\frac{k_{B}}{m}\rho T^{3}\left(\frac{k_{B}}{m}+c_{v}\left(T\right)\right)}q_{i},\\ \frac{{\tilde{\lambda }}_{ll}}{3}=-\frac{\Delta^{K}}{2T^{2}c_{v}^{K}\left(T\right)},\\ {\tilde{\mu }}^{R}=-\frac{\Delta^{R}}{2T^{2}c_{v}^{R}\left(T\right)},\;{\tilde{\mu }}^{V}=-\frac{\Delta^{V}}{2T^{2}c_{v}^{V}\left(T\right)},\\ {\tilde{\lambda }}_{\langle ij\rangle }=\frac{\sigma_{\langle ij\rangle }}{2\frac{k_{B}}{m}\rho T^{2}},\;{\tilde{\mu }}_{i}=-\frac{q_{i}}{2\frac{k_{B}}{m}\rho T^{3}\left(\frac{k_{B}}{m}+c_{v}\left(T\right)\right)}, \end{array} \end{array}$$

where $\Delta^{K}=-\Delta^{R}-\Delta^{V}$ from (15), and $c_{v}^{K,R,V}\left(T\right)=d\varepsilon_{E}^{K,R,V}\left(T\right)/dT$ are the specific heats of the three modes. Inserting (21) and (23) into (19), we can write down the explicit form of the Lagrange multipliers. As is well known, the multipliers coincide with the *main field*, by which the system (12) becomes symmetric hyperbolic. Therefore we heave the well-posed Cauchy problem [9,26,56].

#### 3.6.3. Physical Meaning of the Parameters $\theta^{K}$, $\theta^{R}$, and $\theta^{V}$

In the general framework of RET, nonequilibrium temperatures are defined in terms of the main field [9,53,54]. That is to say, the nonequilibrium temperatures $\vartheta^{K}$, $\vartheta^{R}$, and $\vartheta^{V}$, which are, respectively, related to the translational, rotational, and vibrational modes, are defined as follows:

$$\begin{array}{l} \vartheta^{K}=\frac{3}{2{\hat{\lambda }}_{ll}},\;\vartheta^{R}=\frac{1}{2{\hat{\mu }}^{R}},\;\vartheta^{V}=\frac{1}{2{\hat{\mu }}^{V}}. \end{array}$$

These temperatures coincide with *T* in equilibrium.

In the present case, from (23) and (17), we have the following relations between the nonequilibrium temperatures $\vartheta^{K}$, $\vartheta^{R}$, $\vartheta^{V}$ and the variables $\theta^{K}$, $\theta^{R}$, $\theta^{V}$:

(24) $$\begin{array}{l} \frac{1}{\vartheta^{K}}-\frac{1}{T}=-\frac{\varepsilon_{E}^{K}\left(\theta^{K}\right)-\varepsilon_{E}^{K}\left(T\right)}{T^{2}c_{v}^{K}\left(T\right)},\;\frac{1}{\vartheta^{R}}-\frac{1}{T}=-\frac{\varepsilon_{E}^{R}\left(\theta^{R}\right)-\varepsilon_{E}^{R}\left(T\right)}{T^{2}c_{v}^{R}\left(T\right)},\;\frac{1}{\vartheta^{V}}-\frac{1}{T}=-\frac{\varepsilon_{E}^{V}\left(\theta^{V}\right)-\varepsilon_{E}^{V}\left(T\right)}{T^{2}c_{v}^{V}\left(T\right)}. \end{array}$$

Note that, near equilibrium, we have the approximate relations:

$$\begin{array}{l} \vartheta^{K}=\theta^{K},\;\vartheta^{R}=\theta^{R}\;\vartheta^{V}=\theta^{V}. \end{array}$$

In fact, expanding the right hand sides of the relations (24) with respect to $\theta^{K}-T$, $\theta^{R}-T$, and $\theta^{V}-T$, respectively, we have

$$\begin{array}{l} \frac{1}{\vartheta^{K}}-\frac{1}{T}=-\frac{\theta^{K}-T}{T^{2}},\;\frac{1}{\vartheta^{R}}-\frac{1}{T}=-\frac{\theta^{R}-T}{T^{2}},\;\frac{1}{\vartheta^{V}}-\frac{1}{T}=-\frac{\theta^{V}-T}{T^{2}}. \end{array}$$

Therefore, we can regard $\theta^{K}$, $\theta^{R}$, and $\theta^{V}$ as nonequilibrium temperatures at least not so far from equilibrium, as is the case for the present theory. In general, however, this equivalence no longer holds because the parameters defined through the caloric equation of state depend only on internal energy while the nonequilibrium temperatures defined by the main field may depend on many nonequilibrium quantities. Only in the simple theory that adopts the nonequilibrium internal energies as the dissipative fields such as ET${}_{7}$ [50] and ET${}_{6}$ [53,54], the above equivalence holds generally.

#### 3.6.4. Closure of the Differential System

By using the distribution function (22) with (23), we obtain the constitutive equations for the fluxes up to the first order with respect to the nonequilibrium variables as follows:

$$\begin{array}{l} \begin{array}{l} {\hat{F}}_{ijk}=\int_{R^{3}}\int_{0}^{\infty }\int_{0}^{\infty }mC_{i}C_{j}C_{k}f\;\varphi \left(I^{R}\right)\psi \left(I^{V}\right)\;dI^{R}dI^{V}dC\\ \;\;=\frac{\frac{k_{B}}{m}}{\frac{k_{B}}{m}+c_{v}\left(T\right)}\left(q_{k}\delta_{ij}+q_{j}\delta_{ik}+q_{i}\delta_{jk}\right),\\ {\hat{H}}_{llk}^{R}=\int_{R^{3}}\int_{0}^{\infty }\int_{0}^{\infty }2I^{R}C_{k}f\;\varphi \left(I^{R}\right)\psi \left(I^{V}\right)\;dI^{R}dI^{V}dC\\ \;\;=\frac{2c_{v}^{R}\left(T\right)}{\frac{k_{B}}{m}+c_{v}\left(T\right)}q_{k},\\ {\hat{H}}_{llk}^{V}=\int_{R^{3}}\int_{0}^{\infty }\int_{0}^{\infty }2I^{V}C_{k}f\;\varphi \left(I^{R}\right)\psi \left(I^{V}\right)\;dI^{R}dI^{V}dC\\ \;\;=\frac{2c_{v}^{V}\left(T\right)}{\frac{k_{B}}{m}+c_{v}\left(T\right)}q_{k},\\ {\hat{G}}_{llik}=\int_{R^{3}}\int_{0}^{\infty }\int_{0}^{\infty }\left(mC^{2}+2I^{R}+2I^{V}\right)C_{i}C_{k}f\;\varphi \left(I^{R}\right)\psi \left(I^{V}\right)\;dI^{R}dI^{V}dC\\ \;\;\;=-2\left\{\frac{p{\left(\rho ,T\right)}^{2}}{\rho }-\frac{2}{3}\rho \left(\varepsilon_{E}\left(T\right)+2\frac{p(\rho ,T)}{\rho }\right)\left(\varepsilon_{E}^{K}\left(T\right)+\Delta^{K}\right)\right\}\delta_{ik}-2\left(\varepsilon_{E}\left(T\right)+2\frac{p(\rho ,T)}{\rho }\right)\sigma_{\langle ik\rangle }. \end{array} \end{array}$$

Using the constitutive equations above, we obtain the closed system of field equations for the 15 independent fields, $\rho ,v_{i},T,\Delta^{R},\Delta^{V},\sigma_{\langle ij\rangle },q_{i}$ (the equations of state are given by (9) and (11); the dynamic pressure is given by (16); the production terms will be given below):

(25) $$\begin{array}{l} \frac{\partial \rho }{\partial t}+\frac{\partial }{\partial x_{i}}\left(\rho v_{i}\right)=0,\\ \frac{\partial \rho v_{j}}{\partial t}+\frac{\partial }{\partial x_{i}}\left\{\left[p\left(\rho ,T\right)+\Pi \right]\delta_{ij}-\sigma_{\langle ij\rangle }+\rho v_{i}v_{j}\right\}=0,\\ \frac{\partial }{\partial t}\left\{2\rho \varepsilon_{E}\left(T\right)+\rho v^{2}\right\}+\frac{\partial }{\partial x_{i}}\left\{2q_{i}+2\left[\rho \varepsilon_{E}\left(T\right)+p\left(\rho ,T\right)+\Pi \right]v_{i}-2\sigma_{\langle li\rangle }v_{l}+\rho v^{2}v_{i}\right\}=0,\\ \frac{\partial }{\partial t}\left\{2\rho \left[{\varepsilon_{E}}^{R}\left(T\right)+\Delta^{R}\right]\right\}+\frac{\partial }{\partial x_{i}}\left\{2\rho \left[{\varepsilon_{E}}^{R}\left(T\right)+\Delta^{R}\right]v_{i}+\frac{2c_{v}^{R}\left(T\right)}{\frac{k_{B}}{m}+c_{v}\left(T\right)}q_{i}\right\}={\hat{P}}_{ll}^{R},\\ \frac{\partial }{\partial t}\left\{2\rho \left[{\varepsilon_{E}}^{V}\left(T\right)+\Delta^{V}\right]\right\}+\frac{\partial }{\partial x_{i}}\left\{2\rho \left[{\varepsilon_{E}}^{V}\left(T\right)+\Delta^{V}\right]v_{i}+\frac{2c_{v}^{V}\left(T\right)}{\frac{k_{B}}{m}+c_{v}\left(T\right)}q_{i}\right\}={\hat{P}}_{ll}^{V},\\ \frac{\partial }{\partial t}\left(-\sigma_{\langle ij\rangle }+\rho v_{\langle i}v_{j\rangle }\right)+\frac{\partial }{\partial x_{k}}\left\{\frac{2\frac{k_{B}}{m}}{\frac{k_{B}}{m}+c_{v}\left(T\right)}q_{\langle i}\delta_{j\rangle k}+2\left[p\left(\rho ,T\right)+\Pi \right]v_{\langle i}\delta_{j\rangle k}-\sigma_{\langle ij\rangle }v_{k}-2\sigma_{\langle k\langle i\rangle }v_{j\rangle }+\rho v_{\langle i}v_{j\rangle }v_{k}\right\}={\hat{P}}_{\langle ij\rangle }^{K},\\ \frac{\partial }{\partial t}\left\{2q_{i}+2\left[\rho \varepsilon_{E}\left(T\right)+p\left(\rho ,T\right)+\Pi \right]v_{i}-2\sigma_{\langle li\rangle }v_{l}+\rho v^{2}v_{i}\right\}+\\ \;+\frac{\partial }{\partial x_{k}}\{2\left[p\left(\rho ,T\right)\left(\varepsilon_{E}\left(T\right)+\frac{p(\rho ,T)}{\rho }\right)+\Pi \left(\varepsilon_{E}\left(T\right)+2\frac{p(\rho ,T)}{\rho }\right)\right]\delta_{ik}-2\left[\varepsilon_{E}\left(T\right)+2\frac{p(\rho ,T)}{\rho }\right]\sigma_{\langle ik\rangle }\\ \;\;\;+\frac{2\frac{k_{B}}{m}}{\frac{k_{B}}{m}+c_{v}\left(T\right)}q_{l}v_{l}\delta_{ik}+2\left[1+\frac{\frac{k_{B}}{m}}{\frac{k_{B}}{m}+c_{v}\left(T\right)}\right]\left(q_{i}v_{k}+q_{k}v_{i}\right)+\left[p\left(\rho ,T\right)+\Pi \right]v^{2}\delta_{ik}\\ \;\;\;+2\left[\rho \varepsilon_{E}\left(T\right)+2p\left(\rho ,T\right)+2\Pi \right]v_{i}v_{k}-v^{2}\sigma_{\langle ik\rangle }-2v_{l}v_{i}\delta_{\langle lk\rangle }-2v_{l}v_{k}\sigma_{\langle il\rangle }+\rho v^{2}v_{i}v_{k}\}=2v_{l}{\hat{P}}_{il}^{K}+{\hat{Q}}_{lli}. \end{array}$$

We notice that the field equations of $\rho ,v_{i},T,\sigma_{\langle ij\rangle },q_{i}$ are the same as those obtained by ET${}_{14}$ [19], and the field equation of $\Delta^{K}\left(=-\Delta^{R}-\Delta^{V}\right)$ is essentially the same as the one of the dynamic pressure Π in ET${}_{14}$. However, ET${}_{14}$ treats the contribution from the rotational and vibrational mode of a molecule as a unit, while ET${}_{15}$ has the field equations of $\Delta^{R}$ and $\Delta^{V}$ separately. In this sense, the ET${}_{15}$ theory is a more refined theory than the ET${}_{14}$ theory. Using the ET${}_{15}$ theory, we can analyze the energy exchanges among the three modes.

We can also write down the closed system of field equations in terms of 15 independent fields: $\rho ,v_{i},\theta^{K},\theta^{R},\theta^{V},\sigma_{\langle ij\rangle }$ and $q_{i}$. However, for simplicity, its explicit expression is omitted here.

#### 3.6.5. Entropy Density, Flux and Production

The nonequilibrium part of the entropy density $h-h_{E}$ and the intrinsic entropy flux (non-convective entropy flux) $\varphi_{i}\left(=h_{i}-hv_{i}\right)$ up to the second order with respect to the dissipative fluxes for the truncated system (12) are obtained since we have considered the theory with linear constitutive equations. From (5), inserting the distribution function (22), we have

(26) $$\begin{array}{ll} h-h_{E}= & -\frac{\rho }{2T^{2}}\frac{{\Delta^{K}}^{2}}{c_{v}^{K}\left(T\right)}-\frac{\rho }{2T^{2}}\frac{{\Delta^{R}}^{2}}{c_{v}^{R}\left(T\right)}-\frac{\rho }{2T^{2}}\frac{{\Delta^{V}}^{2}}{c_{v}^{V}\left(T\right)}\\ & -\frac{\sigma_{\langle ik\rangle }\sigma_{\langle ik\rangle }}{4\frac{k_{B}}{m}\rho T^{2}}-\frac{q_{i}q_{i}}{2\frac{k_{B}}{m}\rho T^{3}\left(\frac{k_{B}}{m}+c_{v}\left(T\right)\right)}+O\left(3\right),\\ \varphi_{i}= & \frac{q_{i}}{T}-\frac{2}{2\frac{k_{B}}{m}\rho T^{2}\left(\frac{k_{B}}{m}+c_{v}\left(T\right)\right)}\left(\frac{2\rho }{3}\Delta^{K}q_{i}-q_{j}\sigma_{\langle ij\rangle }\right)+O\left(3\right), \end{array}$$

where O(3) represents the nonequilibrium part composed of terms of order 3 or higher. The evaluation of the higher order term in *h* and $\varphi_{i}$ can be obtained by using the method presented in [57].

The entropy production is consistently determined regardless of the approximation order by exploiting the entropy principle which requires the following form:

(27) $$\begin{array}{l} \Sigma ={\hat{P}}_{ij}^{K}{\tilde{\lambda }}_{ij}+{\hat{P}}_{ll}^{R}{\tilde{\mu }}^{R}+{\hat{P}}_{ll}^{V}{\tilde{\mu }}^{V}+{\hat{Q}}_{lli}{\tilde{\mu }}_{i}+O\left(3\right). \end{array}$$

**Remark** **1.** The O(3) term in h in the first equation of *(26)*, even though being negligibly small, is necessary in the evaluation of the correct entropy production *(27)* up to the second order with respect to the nonequilibrium variables, as is the case for all approximate ET theories near equilibrium.

**Remark** **2.** We have to recall that the entropy, entropy flux *(26)*, and the entropy production *(27)* are not those in *(5)* evaluated by using f of the Boltzmann equation *(1)*, but those for the system of *15* moments evaluated by the approximated distribution function $f_{15}$ given by *(22)*. Because of this reason, *Σ* is not automatically positive as is expected by the H-theorem, but, as in all macroscopic theories, it is necessary to be imposed that *Σ* is non-negative as a consequence of continuum approach of entropy principle.

### 3.7. Production Terms in the Generalized BGK-Model

As seen from (4), the production terms are determined from the collision term in the Boltzmann equation. In this section, for simplicity, we adopt the generalized BGK-model [50], which is the generalization of the collision term utilized in [58,59].

#### 3.7.1. Generalized BGK-Model

The generalized BGK model [50] has three relaxation times corresponding to the following three relaxation processes caused by the molecular collision (see also [4,60,61,62]): (i) Relaxation time $\tau_{K}$ which characterizes the relaxation process of the translational mode (mode *K*) of molecules to its equilibrium state with the distribution function $f_{K:E}$ having the temperature $\theta^{K}$; (ii) Relaxation time $\tau_{bc}$ which characterizes the relaxation process of two of the three modes, that is, mode *K*, rotational mode (mode *R*), and vibrational mode (mode *V*). The state approaches to an equilibrium state of the two modes (b and c mode), characterized by the distribution function $f_{bc:E}$ with a common temperature $\theta^{bc}$. In Table 1, possible three cases are summarized depending on the choice of b and c. We assume $O\left(\tau_{bc}\right)≳O\left(\tau_{K}\right)$. (iii) Relaxation time τ which characterizes the relaxation process in which all modes, *K*, *R* and *V*, approach a local equilibrium state characterized by $f_{E}$ with a common temperature *T* among *K*, *R* and *V* modes. We have the relation: $\tau >\tau_{bc}$.

The generalized BGK collision term with (bc)-process ((bc) = (*KR*), (*KV*), (*RV*)) is proposed as follows [50]:

(28) $$\begin{array}{l} Q^{bc}\left(f\right)=-\frac{1}{\tau_{K}}\left(f-f_{K:E}\right)-\frac{1}{\tau_{bc}}\left(f-f_{bc:E}\right)-\frac{1}{\tau }\left(f-f_{E}\right), \end{array}$$

where the distribution functions $f_{K:E}$ and $f_{bc:E}$ are given as follows:

(29) $$\begin{array}{l} \begin{array}{l} f_{K:E}=\frac{\rho^{RV}\left(I^{R},I^{V}\right)}{m}{\left(\frac{m}{2\pi k_{B}\theta^{K}}\right)}^{3/2}\exp\left(-\frac{mC^{2}}{2k_{B}\theta^{K}}\right)\;\mathrm{with}\;\rho^{RV}\left(I^{R},I^{V}\right)=\int_{R^{3}}mfdc,\\ f_{KR:E}=\frac{\rho^{V}\left(I^{V}\right)}{mA^{R}\left(\theta^{KR}\right)}{\left(\frac{m}{2\pi k_{B}\theta^{KR}}\right)}^{3/2}\exp\left\{-\frac{1}{k_{B}\theta^{KR}}\left(\frac{mC^{2}}{2}+I^{R}\right)\right\}\;\mathrm{with}\;\rho^{V}\left(I^{V}\right)=\int_{R^{3}}\int_{0}^{\infty }mf\;\varphi \left(I^{R}\right)\;dI^{R}dc,\\ f_{KV:E}=\frac{\rho^{R}\left(I^{R}\right)}{mA^{V}\left(\theta^{KV}\right)}{\left(\frac{m}{2\pi k_{B}\theta^{KV}}\right)}^{3/2}\exp\left\{-\frac{1}{k_{B}\theta^{KV}}\left(\frac{mC^{2}}{2}+I^{V}\right)\right\}\;\mathrm{with}\;\rho^{R}\left(I^{R}\right)=\int_{R^{3}}\int_{0}^{\infty }mf\psi \left(I^{V}\right)dI^{V}dc,\\ f_{RV:E}=\frac{\rho }{mA^{R}\left(\theta^{RV}\right)A^{V}\left(\theta^{RV}\right)}{\left(\frac{m}{2\pi k_{B}\theta^{K}}\right)}^{3/2}\exp\left(-\frac{mC^{2}}{2k_{B}\theta^{K}}-\frac{I^{R}+I^{V}}{k_{B}\theta^{RV}}\right). \end{array} \end{array}$$

From (29), we understand that the common temperature of the two modes b and c at every moment is the temperature $\theta^{bc}$, which is determined by the condition:

$$\begin{array}{l} \varepsilon_{E}^{b+c}\left(\theta^{\mathrm{bc}}\right)\equiv \varepsilon_{E}^{b}\left(\theta^{\mathrm{bc}}\right)+\varepsilon_{E}^{c}\left(\theta^{\mathrm{bc}}\right)=\varepsilon_{E}^{b}\left(\theta^{b}\right)+\varepsilon_{E}^{c}\left(\theta^{c}\right). \end{array}$$

The *H*-theorem for this BGK collision term holds as proved in [50].

#### 3.7.2. Production Terms of ET${}_{15}$

In the case of ET${}_{15}$, we obtain the mass densities of equilibrium states of the stage (i) and (ii), $\rho^{RV}$, $\rho^{V}$ and $\rho^{R}$, which appear in (29) as follows:

(30) $$\begin{array}{l} \begin{array}{l} \rho^{RV}\left(I^{R},I^{V}\right)=\rho f_{E}^{\left(R\right)}f_{E}^{\left(V\right)}\left\{1+\frac{\Delta^{R}}{\frac{k_{B}}{m}T^{2}c_{v}^{R}\left(T\right)}\left(\frac{I^{R}}{m}-\varepsilon_{E}^{R}\left(T\right)\right)+\frac{\Delta^{V}}{\frac{k_{B}}{m}T^{2}c_{v}^{V}\left(T\right)}\left(\frac{I^{V}}{m}-\varepsilon_{E}^{V}\left(T\right)\right)\right\},\\ \rho^{V}\left(I^{V}\right)=\rho f_{E}^{\left(V\right)}\left\{1+\frac{\Delta^{V}}{\frac{k_{B}}{m}T^{2}c_{v}^{V}\left(T\right)}\left(\frac{I^{V}}{m}-\varepsilon_{E}^{V}\left(T\right)\right)\right\},\\ \rho^{R}\left(I^{R}\right)=\rho f_{E}^{\left(R\right)}\left\{1+\frac{\Delta^{R}}{\frac{k_{B}}{m}T^{2}c_{v}^{R}\left(T\right)}\left(\frac{I^{R}}{m}-\varepsilon_{E}^{R}\left(T\right)\right)\right\}. \end{array} \end{array}$$

By adopting the generalized BGK model (28) with (29) and (30), we obtain the explicit expressions of the production terms ${\hat{P}}_{ll}^{K}$, ${\hat{P}}_{ll}^{R}$, ${\hat{P}}_{ll}^{V}$, ${\hat{P}}_{\langle ij\rangle }^{K}$ and ${\hat{Q}}_{lli}$. In particular, ${\hat{P}}_{ll}^{K}$, ${\hat{P}}_{ll}^{R}$ and ${\hat{P}}_{ll}^{V}$ depend on the details of the molecular relaxation processes. Inserting (22) with (23) into the corresponding components of (4), the production terms for (bc)-process ((a,b,c) = (*V,K,R*), (*R,K,V*), (*K,R,V*)) are generically expressed as follows:

(31) $$\begin{array}{l} \begin{array}{l} {\hat{P}}_{ll}^{a}=-\frac{2\rho }{\tau }\left(\varepsilon_{E}^{a}\left(\theta^{a}\right)-\varepsilon_{E}^{a}\left(T\right)\right),\\ {\hat{P}}_{ll}^{b}=-\frac{2\rho }{\tau_{\mathrm{bc}}}\left(\varepsilon_{E}^{b}\left(\theta^{b}\right)-\varepsilon_{E}^{b}\left(\theta^{\mathrm{bc}}\right)\right)-\frac{2\rho }{\tau }\left(\varepsilon_{E}^{b}\left(\theta^{b}\right)-\varepsilon_{E}^{b}\left(T\right)\right),\\ {\hat{P}}_{ll}^{c}=-\frac{2\rho }{\tau_{\mathrm{bc}}}\left(\varepsilon_{E}^{c}\left(\theta^{c}\right)-\varepsilon_{E}^{c}\left(\theta^{\mathrm{bc}}\right)\right)-\frac{2\rho }{\tau }\left(\varepsilon_{E}^{c}\left(\theta^{c}\right)-\varepsilon_{E}^{c}\left(T\right)\right),\\ {\hat{P}}_{\langle ij\rangle }=\left(\frac{1}{\tau_{K}}+\frac{1}{\tau_{\mathrm{bc}}}+\frac{1}{\tau }\right)\sigma_{\langle ij\rangle },\\ {\hat{Q}}_{lli}=-2\left(\frac{1}{\tau_{K}}+\frac{1}{\tau_{\mathrm{bc}}}+\frac{1}{\tau }\right)q_{i}. \end{array} \end{array}$$

For the expression (31), it may be useful to introduce the following quantities, i.e., the energy exchanges among the three modes:

$$\begin{array}{l} \delta \equiv \varepsilon_{E}^{b}\left(\theta^{b}\right)-\varepsilon_{E}^{b}\left(\theta^{bc}\right)=-\varepsilon_{E}^{c}\left(\theta^{c}\right)+\varepsilon_{E}^{c}\left(\theta^{bc}\right),\\ \Delta \equiv \Delta^{a}=\varepsilon_{E}^{a}\left(\theta^{a}\right)-\varepsilon_{E}^{a}\left(T\right)=-\varepsilon_{E}^{b+c}\left(\theta^{bc}\right)+\varepsilon_{E}^{b+c}\left(T\right). \end{array}$$

Expanding the energy exchanges with respect to the nonequilibrium temperatures around a temperature *T* up to the first order, we obtain

$$\begin{array}{l} \delta =c_{v}^{b}\left(\theta^{b}-\theta^{bc}\right)=-c_{v}^{c}\left(\theta^{c}-\theta^{bc}\right),\;\Delta =c_{v}^{a}\left(\theta^{a}-T\right)=-c_{v}^{b+c}\left(\theta^{bc}-T\right). \end{array}$$

Here and hereafter we use the notation $c_{v}^{a}$ instead of $c_{v}^{a}\left(T\right)$ and so on for simplicity. Inversely, the nonequilibrium temperatures are expressed as follows:

$$\begin{array}{l} \theta^{a}-T=\frac{\Delta }{c_{v}^{a}},\;\theta^{bc}-T=-\frac{\Delta }{c_{v}^{b+c}},\\ \theta^{b}-T=\frac{\delta }{c_{v}^{b}}-\frac{\Delta }{c_{v}^{b+c}},\;\theta^{c}-T=-\frac{\delta }{c_{v}^{c}}-\frac{\Delta }{c_{v}^{b+c}}. \end{array}$$

The relation between {δ,Δ} and $\left\{\Delta^{b},\Delta^{c}\right\}$ is as follows:

$$\begin{array}{l} \delta =\frac{c_{v}^{c}\Delta^{b}-c_{v}^{b}\Delta^{c}}{c_{v}^{b+c}},\;\Delta =-\Delta^{b}-\Delta^{c}. \end{array}$$

More explicitly, depending on the (bc)-process, this relation is expressed in terms of $\left\{\Delta^{R},\Delta^{V}\right\}$ as summarized in Table 2.

The production terms are now expressed as

$$\begin{array}{l} P_{ll}^{a}=-2\rho \frac{\Delta }{\tau },\;P_{ll}^{b}=-2\rho \frac{\delta }{\tau_{\delta }}+2\rho \frac{c_{v}^{b}}{c_{v}^{b+c}}\frac{\Delta }{\tau },\;P_{ll}^{c}=2\rho \frac{\delta }{\tau_{\delta }}+2\rho \frac{c_{v}^{c}}{c_{v}^{b+c}}\frac{\Delta }{\tau }, \end{array}$$

where $\tau_{\delta }$ is defined by

$$\begin{array}{l} \frac{1}{\tau_{\delta }}\equiv \frac{1}{\tau_{bc}}+\frac{1}{\tau }. \end{array}$$

The system of balance Equations (25) can be rewritten in terms of the independent fields $\left\{\rho ,v_{i},T,\delta ,\Delta ,\sigma_{\langle ij\rangle },q_{i}\right\}$. In particular, we write down it by using the material derivative as follows:

$$\begin{array}{l} \begin{array}{l} \dot{\rho }+\rho \frac{\partial v_{k}}{\partial x_{k}}=0,\\ \rho {\dot{v}}_{i}+\frac{\partial \left\{\left(p+\Pi \right)\delta_{ij}-\sigma_{\langle ij\rangle }\right\}}{\partial x_{j}}=0,\\ \rho c_{v}\dot{T}+\left(p+\Pi \right)\frac{\partial v_{k}}{\partial x_{k}}-\frac{\partial v_{i}}{\partial x_{k}}\sigma_{\langle ik\rangle }+\frac{\partial q_{k}}{\partial x_{k}}=0,\\ \dot{\delta }+\frac{1}{\rho }\left\{A_{1}+\frac{1}{c_{v}}\frac{d}{dT}\left(\frac{c_{v}^{b}}{c_{v}^{b+c}}\right)\Delta \right\}\left\{\left(p+\Pi \right)\delta_{kl}-\sigma_{\langle kl\rangle }\right\}\frac{\partial v_{l}}{\partial x_{k}}+\frac{1}{\rho }\left\{\frac{\frac{k_{B}}{m}A_{1}}{\frac{k_{B}}{m}+c_{v}}+\frac{1}{c_{v}}\frac{d}{dT}\left(\frac{c_{v}^{b}}{c_{v}^{b+c}}\right)\Delta \right\}\frac{\partial q_{k}}{\partial x_{k}}\\ \;+\frac{1}{\rho }\left\{\frac{c_{v}^{b}}{c_{v}^{b+c}}\frac{d}{dT}\left(\frac{\frac{k_{B}}{m}\frac{A_{2}}{c_{v}}+c_{v}^{a}}{\frac{k_{B}}{m}+c_{v}}\right)+\frac{d}{dT}\left(\frac{\frac{k_{B}}{m}\left(1-\frac{A_{2}}{c_{v}}\right)+c_{v}^{b}}{\frac{k_{B}}{m}+c_{v}}\right)\right\}q_{k}\frac{\partial T}{\partial x_{k}}=-\frac{\delta }{\tau_{\delta }},\\ \dot{\Delta }+\frac{1}{\rho }\frac{A_{2}-c_{v}^{a}}{c_{v}}\left\{\left(p+\Pi \right)\delta_{kl}-\sigma_{\langle kl\rangle }\right\}\frac{\partial v_{l}}{\partial x_{k}}+\frac{1}{\rho }\frac{d}{dT}\left(\frac{\frac{k_{B}}{m}\frac{A_{2}}{c_{v}}+c_{v}^{a}}{\frac{k_{B}}{m}+c_{v}}\right)q_{k}\frac{\partial T}{\partial x_{k}}+\frac{1}{\rho }\frac{A_{2}-c_{v}^{a}}{\left(\frac{k_{B}}{m}+c_{v}\right)c_{v}}\frac{\partial q_{k}}{\partial x_{k}}=-\frac{\Delta }{\tau },\\ {\dot{\sigma }}_{\langle ij\rangle }-2\left(p+\Pi \right)\frac{\partial v_{\langle i}}{\partial x_{j\rangle }}+\sigma_{\langle ij\rangle }\frac{\partial v_{k}}{\partial x_{k}}+2\frac{\partial v_{\langle i}}{\partial x_{k}}\sigma_{\langle j\rangle k\rangle }+\frac{2\frac{k_{B}}{m}}{{\left(\frac{k_{B}}{m}+c_{v}\right)}^{2}}\frac{dc_{v}}{dT}\frac{\partial T}{\partial x_{k}}q_{\langle i}\delta_{j\rangle k}-\frac{2\frac{k_{B}}{m}}{\frac{k_{B}}{m}+c_{v}}\frac{\partial q_{\langle i}}{\partial x_{j\rangle }}=-\left(\frac{1}{\tau_{K}}+\frac{1}{\tau_{\delta }}\right)\sigma_{\langle ij\rangle },\\ {\dot{q}}_{i}+\frac{2\frac{k_{B}}{m}+c_{v}}{\frac{k_{B}}{m}+c_{v}}q_{i}\frac{\partial v_{k}}{\partial x_{k}}+\frac{\frac{k_{B}}{m}}{\frac{k_{B}}{m}+c_{v}}q_{k}\frac{\partial v_{k}}{\partial x_{i}}+\frac{2\frac{k_{B}}{m}+c_{v}}{\frac{k_{B}}{m}+c_{v}}q_{k}\frac{\partial v_{i}}{\partial x_{k}}+\left\{\left(\frac{k_{B}}{m}+c_{v}\right)p\delta_{ki}+\left(2\frac{k_{B}}{m}+c_{v}\right)\left(\Pi \delta_{ki}-\sigma_{\langle ki\rangle }\right)\right\}\frac{\partial T}{\partial x_{k}}\\ \;-\frac{p}{\rho }\frac{\partial p}{\partial x_{i}}+\frac{1}{\rho }\left\{\left(p-\Pi \right)\delta_{ki}+\sigma_{\langle ki\rangle }\right\}\frac{\partial }{\partial x_{l}}\left\{\left(p+\Pi \right)\delta_{kl}-\sigma_{\langle kl\rangle }\right\}=-\left(\frac{1}{\tau_{K}}+\frac{1}{\tau_{\delta }}\right)q_{i}, \end{array} \end{array}$$

where $A_{1}$, $A_{2}$ and Π are given in Table 3.

It is possible to verify that the present production terms give a non-negative entropy production (27) (Σ≥0). In fact, we have

$$\begin{array}{l} \Sigma =\frac{\rho }{\tau T^{2}}\frac{c_{v}}{c_{v}^{a}c_{v}^{b}+c}\Delta^{2}+\frac{\rho }{\tau_{\delta }T^{2}}\frac{c_{v}^{b+c}}{c_{v}^{b}c_{v}^{c}}\delta^{2}+\frac{1}{2pT}\left(\frac{1}{\tau_{K}}+\frac{1}{\tau_{\delta }}\right)\left(\sigma_{\langle ij\rangle }\sigma_{\langle ij\rangle }+\frac{2}{T\left(\frac{k_{B}}{m}+c_{v}\right)}q_{i}q_{i}\right)\geq 0, \end{array}$$

since the relaxation times and specific heats are positive. Therefore the entropy principle is satisfied in the present ET${}_{15}$ theory.

### 3.8. Maxwellian Iteration and Phenomenological Coefficients: Shear and Bulk Viscosities, and Heat Conductivity

To obtain the system of the NSF theory, we carry out the Maxwellian iteration [63] on (25) and retain the first order terms with respect to the relaxation times τ, $\tau_{\sigma }$ and $\tau_{q}$. Then we have

$$\begin{array}{l} \delta =-\tau_{\delta }\frac{p}{\rho }A_{1}\frac{\partial v_{i}}{\partial x_{i}},\;\Delta =-\tau \frac{p}{\rho }\frac{A_{2}-c_{v}^{a}}{c_{v}}\frac{\partial v_{i}}{\partial x_{i}},\;\sigma_{\langle ij\rangle }=2p\tau_{\sigma }\frac{\partial v_{\langle i}}{\partial x_{j\rangle }},\;q_{i}=-\left(\frac{k_{B}}{m}+c_{v}\right)p\tau_{q}\frac{\partial T}{\partial x_{i}}, \end{array}$$

where, from (31),

$$\begin{array}{l} \frac{1}{\tau_{\sigma }}=\frac{1}{\tau_{q}}=\frac{1}{\tau_{K}}+\frac{1}{\tau_{\delta }}\; \end{array}$$

If $\tau_{K}\ll \tau_{\mathrm{bc}}$, we have the relation: $\tau_{\sigma }$ = $\tau_{q}$∼$\tau_{K}$. In particular, for (bc)-process ((bc)=(KR) or (KV)), we have

$$\begin{array}{l} \Pi =\Pi^{bc}+\Pi^{a}\;\mathrm{with}\\ \Pi^{bc}=p\left(\rho ,\theta^{K}\right)-p\left(\rho ,\theta^{bc}\right)=-\tau_{\delta }p\frac{k_{B}}{m}\frac{c_{v}^{c}}{c_{v}^{b}c_{v}^{b+c}}\frac{\partial v_{i}}{\partial x_{i}}\\ \Pi^{a}=p\left(\rho ,\theta^{bc}\right)-p\left(\rho ,T\right)=-\tau p\frac{k_{B}}{m}\frac{c_{v}^{a}}{c_{v}^{b+c}c_{v}}\frac{\partial v_{i}}{\partial x_{i}}, \end{array}$$

and for (RV)-process, we have

$$\begin{array}{l} \Pi =-\tau p\frac{k_{B}}{m}\frac{c_{v}^{R+V}}{c_{v}^{K}c_{v}}\frac{\partial v_{i}}{\partial x_{i}}. \end{array}$$

Recalling the definition of the bulk viscosity ν, shear viscosity μ, and heat conductivity κ:

$$\begin{array}{l} \Pi =-\nu \frac{\partial v_{i}}{\partial x_{i}},\;\sigma_{\langle ij\rangle }=2\mu \frac{\partial v_{\langle i}}{\partial x_{j\rangle }},\;q_{i}=-\kappa \frac{\partial T}{\partial x_{i}}, \end{array}$$

we obtain

(32) $$\begin{array}{l} \begin{array}{l} \nu =\left\{\begin{array}{cc} \frac{k_{B}}{m}\frac{c_{v}^{c}}{c_{v}^{b}c_{v}^{b+c}}p\tau_{\delta }+\frac{k_{B}}{m}\frac{c_{v}^{a}}{c_{v}^{b+c}c_{v}}p\tau & \mathrm{for}\;\left(bc\right)-\mathrm{process}\;((bc)=(\mathrm{KR})\;\mathrm{or}\;(\mathrm{KV}))\\ \frac{k_{B}}{m}\frac{c_{v}^{R+V}}{c_{v}^{K}c_{v}}p\tau & \mathrm{for}\;(\mathrm{RV})-\mathrm{process} \end{array}\right.,\\ \mu =p\tau_{\sigma },\\ \kappa =\left(\frac{k_{B}}{m}+c_{v}\right)p\tau_{q}. \end{array} \end{array}$$

It should be noted that, as usual in the BGK model, the Prandtl number predicted by the present model is not satisfactory. One possible way to avoid this difficulty is that we use the procedure adopted in the phenomenological ET theory of rarefied polyatomic gases. That is, the relaxation times τ, $\tau_{\delta }$, $\tau_{\sigma }$, and $\tau_{q}$ are regarded as functions of ρ and *T* that are estimated by using the experimental data on ν, μ and κ. In this respect, for (*RV*)-process, the relations (32) are the same as those derived by ET${}_{14}$ [19]. On the other hand, for (bc)-process ((bc)=(*KR*) or (*KV*)), only the relation for ν is different from the one derived by $ET_{14}$.

## 4. Summary and Outlook

In the present paper, firstly, a brief review on the present status of RET of gases was presented. Secondly, the molecular ET theory of rarefied polyatomic gases with 15 independent fields has been developed. By adopting the generalized BGK-type collision term, we have obtained the closed system of field equations explicitly, where detailed energy exchange among the different modes has been taken into account appropriately. The relaxation times introduced in the theory can be estimated by the experimental data on the shear and bulk viscosities and heat conductivity. It was shown that the NSF theory is derived from the RET theory as a limiting case of small relaxation times.

With this theory, we can explore highly nonequilibrium phenomena beyond the local equilibrium where the NSF theory is no longer valid. In particular, we are now studying linear wave dispersion relation, shock wave, and light scattering by comparing the theoretical predictions with experimental data. These results will soon be reported.

## Figures and Tables

**Table 1:** Three possible relaxation processes in the second stage (ii).

(bc)-Process	(a,b,c)	Relaxation Time	Collision Term
(KR)-process	(V,K,R)	$\tau_{KR}$	$Q^{KR}\left(f\right)$
(KV)-process	(R,K,V)	$\tau_{KV}$	$Q^{KV}\left(f\right)$
(RV)-process	(K,R,V)	$\tau_{RV}$	$Q^{RV}\left(f\right)$

**Table 2:** Relation between {δ,Δ} and $\left\{\Delta^{R},\Delta^{V}\right\}$.

(bc)	δ	Δ
(KR)	$-\frac{c_{v}^{R}}{c_{v}^{K+R}}\Delta^{V}-\Delta^{R}$	$\Delta^{V}$
(KV)	$-\frac{c_{v}^{V}}{c_{v}^{K+V}}\Delta^{R}-\Delta^{V}$	$\Delta^{R}$
(RV)	$\frac{c_{v}^{V}\Delta^{R}-c_{v}^{R}\Delta^{V}}{c_{v}^{R+V}}$	$-\Delta^{R}-\Delta^{V}$

**Table 3:** Explicit expression of $A_{1}$, $A_{2}$ and Π.

(bc)	$A_{1}$	$A_{2}$	Π
(KR) or (KV)	$\frac{c_{v}^{c}}{c_{v}^{b+c}}$	0	$\frac{2}{3}\rho \left(\delta -\frac{c_{v}^{b}}{c_{v}^{b+c}}\Delta \right)$
(RV)	0	$c_{v}$	$\frac{2}{3}\rho \Delta$
